# Wrist Accelerometer Estimates of Physical Activity Intensity During Walking in Older Adults and People Living With Complex Health Conditions: Retrospective Observational Data Analysis Study

**DOI:** 10.2196/41685

**Published:** 2023-03-15

**Authors:** Kyle S Weber, F Elizabeth Godkin, Benjamin F Cornish, William E McIlroy, Karen Van Ooteghem

**Affiliations:** 1 Department of Kinesiology and Health Sciences University of Waterloo Waterloo, ON Canada

**Keywords:** neurodegenerative disease, aging, older adults, wearable sensors, physical activity, activity intensity, activity monitoring, exercise prescription, accelerometry, health technology

## Abstract

**Background:**

Accurate measurement of daily physical activity (PA) is important as PA is linked to health outcomes in older adults and people living with complex health conditions. Wrist-worn accelerometers are widely used to estimate PA intensity, including walking, which composes much of daily PA. However, there is concern that wrist-derived PA data in these cohorts is unreliable due to slow gait speed, mobility aid use, disease-related symptoms that impact arm movement, and transient activities of daily living. Despite the potential for error in wrist-derived PA intensity estimates, their use has become ubiquitous in research and clinical application.

**Objective:**

The goals of this work were to (1) determine the accuracy of wrist-based estimates of PA intensity during known walking periods in older adults and people living with cerebrovascular disease (CVD) or neurodegenerative disease (NDD) and (2) explore factors that influence wrist-derived intensity estimates.

**Methods:**

A total of 35 older adults (n=23 with CVD or NDD) wore an accelerometer on the dominant wrist and ankle for 7 to 10 days of continuous monitoring. Stepping was detected using the ankle accelerometer. Analyses were restricted to gait bouts ≥60 seconds long with a cadence ≥80 steps per minute (LONG walks) to identify periods of purposeful, continuous walking likely to reflect moderate-intensity activity. Wrist accelerometer data were analyzed within LONG walks using 15-second epochs, and published intensity thresholds were applied to classify epochs as sedentary, light, or moderate-to-vigorous physical activity (MVPA). Participants were stratified into quartiles based on the percent of walking epochs classified as sedentary, and the data were examined for differences in behavioral or demographic traits between the top and bottom quartiles. A case series was performed to illustrate factors and behaviors that can affect wrist-derived intensity estimates during walking.

**Results:**

Participants averaged 107.7 (SD 55.8) LONG walks with a median cadence of 107.3 (SD 10.8) steps per minute. Across participants, wrist-derived intensity classification was 22.9% (SD 15.8) sedentary, 27.7% (SD 14.6) light, and 49.3% (SD 25.5) MVPA during LONG walks. All participants measured a statistically lower proportion of wrist-derived activity during LONG walks than expected (all *P*<.001), and 80% (n=28) of participants had at least 20 minutes of LONG walking time misclassified as sedentary based on wrist-derived intensity estimates. Participants in the highest quartile of wrist-derived sedentary classification during LONG walks were significantly older (*t*_16_=4.24, *P*<.001) and had more variable wrist movement (*t*_16_=2.13, *P*=.049) compared to those in the lowest quartile.

**Conclusions:**

The current best practice wrist accelerometer method is prone to misclassifying activity intensity during walking in older adults and people living with complex health conditions. A multidevice approach may be warranted to advance methods for accurately assessing PA in these groups.

## Introduction

Regular physical activity (PA) is important for minimizing the risk of adverse health outcomes and premature mortality [1,2]. The many benefits of PA extend to older adults and those living with complex health conditions such as cerebrovascular disease (CVD) or neurodegenerative disease (NDD). For example, routine PA over the lifespan may mitigate age-related cognitive decline [3], and engagement in regular PA may positively impact motor and nonmotor symptoms of NDDs [4-7]. Published movement guidelines recommend participating in a minimum of 150 minutes of moderate-to-vigorous physical activity (MVPA) per week (≥ 3 metabolic equivalents of task) [2,8], several hours of light PA (1.5-3.0 metabolic equivalents of task) per week, and limiting sedentary time [8]. Walking is the most commonly reported leisure time activity [9], and across the lifespan, preferred pace walking is classified as a moderate-intensity aerobic activity [10,11]. Thus, it is critically important to accurately quantify walking behavior to describe overall activity level and further understand the relationship between PA and health outcomes in older adults and people living with complex health conditions.

Accelerometry is commonly used to measure naturally occurring activity [12] and its relationship with other health-related behaviors such as sleep and sedentary behavior [8]. Accelerometry affords the opportunity to move beyond the constraints of laboratory or clinical measures and overcome challenges associated with self-report assessments of PA by objectively measuring PA intensity in free-living, ecologically valid settings [13]. Free-living activity assessment is typically conducted using an accelerometer placed on the wrist, thigh, or hip [14-20]. While wrist-worn devices are convenient and well-tolerated due to comfort and ease of use [21-23], there are several circumstances when the approach may be susceptible to error in estimating activity volume and intensity, including instances of walking. Specifically, isolated arm movement (eg, gesticulation), absence of arm movement (eg, using a mobility aid such as a walker), or activities of daily living (ADLs; eg, pushing a shopping cart) may result in over- or underestimation of activity intensity [24]. Disease- or age-related changes in arm movement, including variable arm swing, limb asymmetry or impaired limb coordination, and reduced arm swing during slow gait [25-27], can also impact the relationship between arm motion and walking, leading to a dissociation between wrist-based estimates of intensity and the actual energy requirements of the activity [28]. Given the potential susceptibility to error and the ubiquitous use of wrist-worn accelerometry for PA intensity classification in research and clinical applications, there is an important need to confront the limitations of wrist-derived PA intensity estimates during periods of unconstrained, free-living walking.

A viable candidate for advancing PA intensity estimation is the concurrent use of an ankle-worn device. The ankle provides an alternative wear location that robustly detects walking and other lower limb or whole-body activities such as cycling [29,30] and accurately measures the spatiotemporal characteristics used to evaluate gait control [31-33]. Compared to wrist- or hip-mounted accelerometers, ankle-worn accelerometers provide more accurate results at slower gait speeds, which are often exhibited by older adults and people living with complex health conditions [34]. Multidevice approaches (eg, combining wrist- and ankle-worn devices) have the potential to improve PA estimates by providing necessary context and the opportunity to resolve discrepancies [35]. For example, obtaining a profile of daily walking behavior from ankle accelerometer data provides windows of known activity that can be compared against other indices of activity, such as wrist-based estimates of intensity. This approach has the potential to improve the accuracy of PA estimates and can be a useful model to reveal the dissociation in activity classification based on wear location.

This study examined the estimated activity intensity from a wrist-worn accelerometer during walking as identified from synchronous data captured at the ankle in an extended free-living collection. The primary objective of this study was to determine the accuracy of PA intensity measured from the wrist when compared to known periods of walking (from the ankle) in older adults and people living with CVD or a range of NDDs. It was hypothesized that PA intensity derived from wrist accelerometry will underestimate the intensity of walking within these cohorts, including instances when known ankle-derived walking bouts are misclassified as sedentary behavior. A secondary objective was to explore demographic, behavioral, and disease-related characteristics that contribute to variability in measured intensity from wrist-worn accelerometers during known walking bouts in free-living conditions.

## Methods

### Participants

Participants were drawn from 2 studies conducted by the Ontario Neurodegenerative Disease Research Initiative (ONDRI): the Remote Monitoring in Neurodegenerative Disease (ReMiNDD) study [23] and the Health in Aging and Neurodegenerative Diseases and DementiaS in Ontario (HANDDS-ONT) study. Given the specific interest in aging and disease, this study included participants who were 65 years of age or older. Participants in the ReMiNDD study met standard clinical diagnostic criteria for CVD or 1 of 4 NDDs, including Alzheimer disease or amnestic mild cognitive impairment (AD/MCI), frontotemporal dementia (FTD), Parkinson disease (PD), and amyotrophic lateral sclerosis (ALS; all referred to as older adults living with an NDD (“OA-NDD”)) [23,36]. Participants in the HANDDS-ONT study included adults who self-reported meeting clinical criteria for possible or probable CVD, AD/MCI, FTD, PD, or ALS (also referred to as “OA-NDD”) or those who lived independently in the community with no clinical diagnosis of an NDD (referred to as older adults (“OA”) within this study).

### Ethics Approval

The ReMiNDD and HANDDS-ONT studies were both approved by the Sunnybrook Health Sciences research ethics board (No: 1832 and CTO No: 3589, respectively), and all participants provided written informed consent prior to data collection. All study data were deidentified. Participants did not receive compensation for their study participation.

### Procedures

#### Overview

Data were collected between May 2019 and March 2020 for the ReMiNDD study (see [23] for a detailed protocol), with HANDDS-ONT study data collection ongoing since August 2021 [37]. Briefly, both studies began with a baseline visit that involved the collection of medical history and instrumentation with multiple wearable devices that participants wore over the course of a 7-day (ReMiNDD) or up to 10-day (HANDDS-ONT) collection period. Participants also completed a cognitive assessment and questionnaires about their health and disease status (see section “Demographics and Clinical Measures”). At the end of the collection period, wearable devices were returned to the study team for data offloading, processing, and analysis.

#### Data Collection

##### Demographic and Clinical Measures

Participants self-reported demographic, health history, gait aid use, and hand dominance information for both studies. A trained member of the study team administered the Montreal Cognitive Assessment (MoCA) [38] and the modified Rankin Scale (mRS) [39], with the latter only being administered as a part of the ReMiNDD study and for HANDDS participants living with CVD. This information was collected and stored using REDCap electronic case report forms [40] and hosted on Brain-CODE [41-43].

##### Wearables Data

For the ReMiNDD study, GENEActiv Original accelerometers (ActivInsights) were worn on both wrists and ankles, sampling at a frequency of 75 Hz. For the HANDDS-ONT study, Axivity 6 inertial measurement units (AX6, Axivity Ltd) containing an accelerometer were worn on the participants’ self-reported dominant or least impaired wrist and an ankle (ipsilateral for all but n=1), sampling at a frequency of 50 Hz. Both devices output raw triaxial acceleration, which allows data processing consistent with that of other works without concern for the use of proprietary preprocessing or different device types or sampling rates [44]. Following collection, data were offloaded to a secure network drive and processed using custom analytics implemented in Python, as described in the Data Processing and Analysis section below. Only data from the self-reported dominant or least impaired side were used in this study.

#### Data Processing and Analysis

Custom Python software was used for all data processing and analysis. GENEActiv and Axivity files were converted to a standardized data format (European Data Format). Wrist and ankle data were synchronized using either the clock drift calculation (GENEActiv) or synchronization events performed manually during the collection period (AX6). During stationary periods, accelerometer calibration was evaluated using gravity as a constant, known acceleration value, and corrected as necessary [45]. Analytics were run to detect device nonwear [46], periods of sleep (wrist device; [47]), steps (ankle device; unpublished), and activity using thresholds for intensity (“cut points”) developed in a sample of older adults (mean 77, SD 5 years; wrist device; [44]). These cut points use average vector magnitude (AVM) data, which are derived using low-pass filtered triaxial accelerometer data and are independent of sampling rates, and therefore can be applied to both processed GENEActiv and Axivity data. To classify total free-living wrist-derived activity volumes, only full calendar days with at least 10 hours of device wear during waking hours were included (considered “valid days”), consistent with many studies of PA [48].

Using the steps detected by the ankle accelerometer, the data set was annotated with bouts of walking that served to anchor wrist-based activity estimates. Walking bouts had a maximum resting period of 5 seconds [49], a median cadence above 80 steps per minute (spm), and a minimum duration of 60 seconds (referred to as LONG walks) [50]. These criteria were selected to identify bouts of purposeful, continuous walking assumed to reflect moderate-intensity activity [28].

To derive PA estimates from the wrist during known periods of walking, wrist data were first checked for periods of nonwear [46]. One-second epochs were generated using the AVMs of the calibrated, gravity-subtracted triaxial accelerometer wrist data. Epoched wrist data were then reaveraged into 15-second epochs, and epochs that ended after the end of their associated LONG walk were not included in the analysis of PA intensity. The wrist-derived intensity was calculated for all 15-second epochs during LONG walks using published activity cut points for older adults [44]. These cut points differentiate between sedentary, light, and moderate intensity. With no cut-point to differentiate moderate from vigorous intensity, epochs above the moderate-intensity threshold were classified as MVPA. For each intensity category, activity volume is expressed as a percentage of LONG walk epochs.

To examine the clinical implications of misclassifying wrist-derived activity intensity, wrist-derived sedentary minutes during LONG walks were totaled per participant, and 20 minutes of sedentary time was set as a clinically important threshold based on published movement guidelines [8]. These guidelines recommend 150 minutes of MVPA per week, which equates to approximately 20 minutes of MVPA per day. As the collection periods were approximately 7 days long, the misclassification of 20 minutes of MVPA as sedentary time represents approximately one day’s worth of recommended MVPA.

### Statistical Analyses

Participant demographic and clinical profiles, as well as device data volume and daily gait bout characteristics, were summarized using descriptive statistics (mean, SD; range). To address the primary objective of determining the accuracy of wrist-based activity estimates during walking, epochs were dichotomized into sedentary or active classifications (light and MVPA intensities grouped). Combining light and MVPA into 1 “active” category was done to provide a conservative analysis of how often wrist-based methods underestimate PA intensity by requiring walking bouts to be labeled “sedentary” to be considered misclassified. A Fisher exact test (conducted using scipy.stats [51]) was conducted for each participant to determine if the proportion of walking epochs classified as active (light or MVPA) during LONG walks, based on wrist-derived intensity estimates, was different than the expected proportion that all LONG walking epochs (walking bouts defined using step detection from the ankle accelerometer) should be classified as active. A Bonferroni correction was applied to account for multiple comparisons.

To address the secondary objective of examining potential demographic or behavioral characteristics that affect wrist-derived activity estimates, participants were stratified into quartiles based on the percent of epochs during LONG walks classified as sedentary. The first and fourth quartiles were used to compare those with the lowest percent of sedentary epochs during LONG walks (SED_Q1) to those with the highest (SED_Q4), yielding 2 groups of 9 participants. Two-tailed independent *t* tests (conducted using Pingouin [52]) were used to test the between-group differences in age, number of long walks, length of time spent walking, cadence, and wrist AVM variability. The statistical significance was set at α=.05.

In addition to quantifying and characterizing the dissociation between ankle- and wrist-based intensity at the group level, the data were explored for factors that could impact variability in wrist-derived intensity estimates observed within and across participants, including functional capacity, arm-dominant behaviors, and symptoms of the disease. Representative data from 3 participants were extracted as a series of case examples based on their percent of wrist-derived sedentary classification during LONG walks and included: a participant near the 50th percentile of all OA participants (OA6), the participant with the highest percent of sedentary classification among gait aid users (FTD1), and a participant from the NDD cohort who presented with the highest level of functional disability as measured via the mRS score (PD1). Illustrative data segments were chosen for each case example based on a visual inspection of the walking bouts included in the primary analysis (LONG walks).

## Results

### Participant Characteristics

A total of 35 participants were included in this study (n=20 from ReMiNDD and n=15 from HANDDS-ONT), represented as follows: 12 OA and 6 PD, 9 AD/MCI, 6 CVD, 1 FTD, and 1 ALS (n=23 OA-NDD). Participants were 65 to 87 (mean 73.1, SD 5.8) years of age, and 43% (15/35) of the participants were female. The average MoCA score was 25.0 (SD 2.6; range 17-30; n=31), and the mRS was 1.7 (SD 1.0; range 0-3; n=20). Two participants used a gait aid, including a walker (n=1; 1 FTD) and a cane (n=1; 1 PD). Table 1 summarizes the demographic and clinical characteristics of participants overall and when separated into the OA and OA-NDD cohorts.

**Table 1:** Participant characteristics.

	OA^a^ cohort (n=12)	OA-NDD^b^ cohort (n=23)	Overall cohort (n=35)
Age (years), mean (SD)	74.1 (7.5)	72.6 (4.8)	73.1 (5.8)
Sex: female, n (%)	8 (67)	7 (30)	15 (43)
Right-handed, n (%)	11 (92)	22 (96)	33 (94)
Use of a gait aid, n (%)	0 (0)	2 (9)	2 (6)
MoCA^c^ (0-30)^d^ (n=31), mean (SD)	24.8 (2.1)	25.2 (2.9)^e^	25.0 (2.6)
mRS^f^ (0-5)^g^ (n=20), mean (SD)	N/A^h^	1.7 (1.0)	1.7 (1.0)

^a^OA: older adults without a neurodegenerative disease diagnosis.

^b^OA-NDD: older adults living with a neurodegenerative disease.

^c^MoCA: Montreal Cognitive Assessment.

^d^Higher score indicates greater functioning.

^e^Missing n=4 responses (n=1 missing; participant unable to complete MoCA due to cognitive impairment).

^f^mRS: modified Rankin Scale.

^g^Lower score indicates greater functioning.

^h^N/A: not applicable.

### Summary of Overall Daily Activity (Wrist-Derived) and Daily Walking (Ankle-Derived)

After accounting for device nonwear, sleep, and partial collection days, participants produced 7.1 (SD 1.2; range 3-9) valid days with 15.0 (SD 1.3; range 11.9-17.7) hours of data during waking hours on these days. On average, participants were sedentary for 776 (SD 70; range 598-938) minutes per day, performed 67 (SD 25; range 25-116) minutes of light activity per day, and performed 58 (SD 36; range 7-151) minutes of MVPA per day based on wrist intensity estimates. When normalized to the amount of valid data, participants spent 86% (SD 4; range 75-95) of their time sedentary, 7% (SD 3; range 3-13) of their time in light activity, and 6% (SD 4; range 1-15) of their time in MVPA. Participants accumulated an average of 8864 (SD 3261; range 3792-15154) total steps per day, inclusive of all walking bouts measured using ankle accelerometry (a minimum of 5 steps; ALL walks; unpublished data).

There were a total of 3768 walking bouts that met the criteria for inclusion (≥60 seconds with a median cadence above 80 spm) in the primary analysis (LONG walks). On average, participants had 107.7 (SD 55.6; range 10-250) LONG walks. Participants accumulated a total of 258.9 (SD 145.1; range 29.3-518.8) walking minutes in LONG walks. As a percentage of ALL walks, 28.2% (SD 13.5; range 4.1-57.3) of the total walking time was included in the LONG walks. Median cadence during LONG walks was 107.3 (SD 10.9; range 89.4-135.1) spm.

### PA Intensity Classification (Wrist-Derived) During LONG Walks

**Figure 1:**
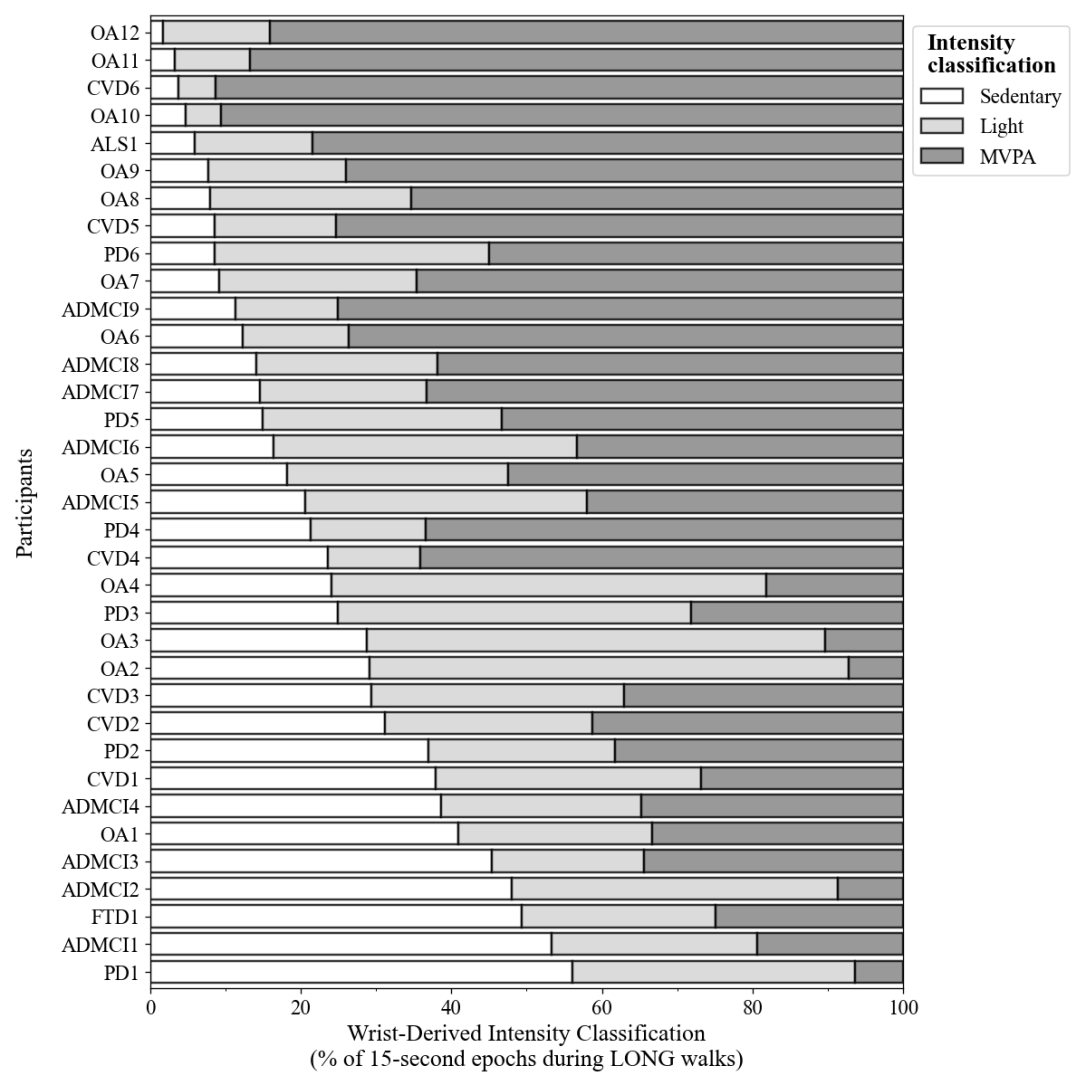
Wrist-derived intensity classification during LONG walks for each participant. Distribution of wrist-derived sedentary (white), light (light gray), and moderate-to-vigorous physical activity (dark gray) intensity epochs. LONG walks were detected from ankle sensor data. Each row is a single participant, with wrist-derived activity volumes totaling 100%. AD/MCI: Alzheimer disease or amnestic mild cognitive impairment; ALS: amyotrophic lateral sclerosis; CVD: cerebrovascular disease; FTD: frontotemporal dementia; MVPA: moderate-to-vigorous physical activity; OA: older adults without a neurodegenerative disease diagnosis; PD: Parkinson’s disease.

Each participant’s volume of wrist-derived activity intensity during LONG walks is illustrated in Figure 1. Across all walking epochs during LONG walks (n=36,248), 20.54% (n=7446) were classified as sedentary, 24.44% (n=8861) were classified as light activity, and 55.01% (n=19,941) were classified as MVPA. Across participants, wrist-derived activity intensity classification during LONG walks was 22.9% (SD 15.8; range 1.7-56.4) sedentary, 27.7% (SD 14.6; range 4.8-63.7) light activity, and 49.3% (SD 25.5; range 6.4-91.3) MVPA. For all participants, OA and OA-NDD, the proportion of measured wrist activity intensities during LONG walks differed significantly from the expected distribution of only active epochs (all *P*<.001). Furthermore, 80% of the participants (28/35) had at least 20 minutes of walking time misclassified as sedentary based on wrist intensity estimates, which exceeded the established threshold for clinically meaningful impact on measured daily PA.

### Identification of Factors Related to Variability in Wrist-Derived Activity Intensity Estimates

#### Group Level Comparison

Differences in demographics, walking behavior, and activity volumes between groups with the lowest percent wrist-derived sedentary classification during LONG walks (SED_Q1) and the highest (SED_Q4) are summarized in Table 2. On average, the participants in SED_Q4 were significantly older (*t*_16_=4.24, *P*<.001) and had a higher wrist AVM coefficient of variation (CoV; *t*_16_=2.13, *P*=.049) than the participants in SED_Q1. There were no significant differences in the number of LONG walks (*t*_16_=0.03, *P*=.98), time accumulated in LONG walks (*t*_16_=2.01, *P*=.06), or median cadence (*t*_16_=1.26, *P*=.23). There were 8 people living with NDD and 1 gait aid user (FTD) in SED_Q4 compared to 4 people living with NDD and no gait aid users in SED_Q1.

Data are presented as mean (SD) unless otherwise noted.

**Table 2:** Comparing demographic characteristics, walking behavior, and daily mean activity levels of the group with the lowest (SED_Q1) to the group with the highest (SED_Q4) percent of wrist-derived sedentary classification detected during LONG walks. *P* values from unpaired t tests between quartile groups (all df=16).

	Characteristics	SED_Q1	SED_Q4	*P* value
Gait aid use, n		0	1	N/A^a^
Age (years)		69.0 (2.8)	76 (4.5)	<.001
Cohorts				
	OA^b^, n	5	1	N/A
	AD/MCI^c^, n	0	4	N/A
	PD^d^, n	1	2	N/A
	CVD^e^, n	2	1	N/A
	ALS^f^, n	1	0	N/A
	FTD^g^, n	0	1	N/A
Walking behavior				
	Total time in LONG walks (min)	338.5 (156.1)	218.9 (86.0)	.06
	Percent LONG walk epochs classified as sedentary (% sedentary)	5.8 (2.6)	45.2 (7.0)	<.001
	AVM CoV^h^ (%)	37.5 (16.9)	51.2 (9.4)	.049
	Median cadence (steps/min)	112.8 (10.7)	105.8 (12.7)	.23
Daily mean activity levels				
	Sedentary (hours)	12.6 (1.9)	12.7 (1.3)	.89
	Sedentary (% valid time)	82.4 (7.0)	89.5 (3.5)	.015
	Light (min)	75.1 (27.3)	59.5 (23.1)	.21
	MVPA^i^ (min)	85.4 (37.3)	33.7 (19.6)	.002
	Step count	10355 (3615)	7974 (1882)	.10
	Detected sleep (hours)	7.0 (1.2)	7.9 (1.7)	.21

^a^N/A: not applicable.

^b^OA: older adults without a neurodegenerative disease diagnosis.

^c^AD/MCI: Alzheimer disease or amnestic mild cognitive impairment.

^d^PD: Parkinson disease.

^e^CVD: cerebrovascular disease.

^f^ALS: amyotrophic lateral sclerosis.

^g^FTD: frontotemporal dementia.

^h^AVM CoV*:* average vector magnitude coefficient of variation.

^i^MVPA: moderate-to-vigorous physical activity.

#### Case Examples

##### Overview

**Figure 2:**
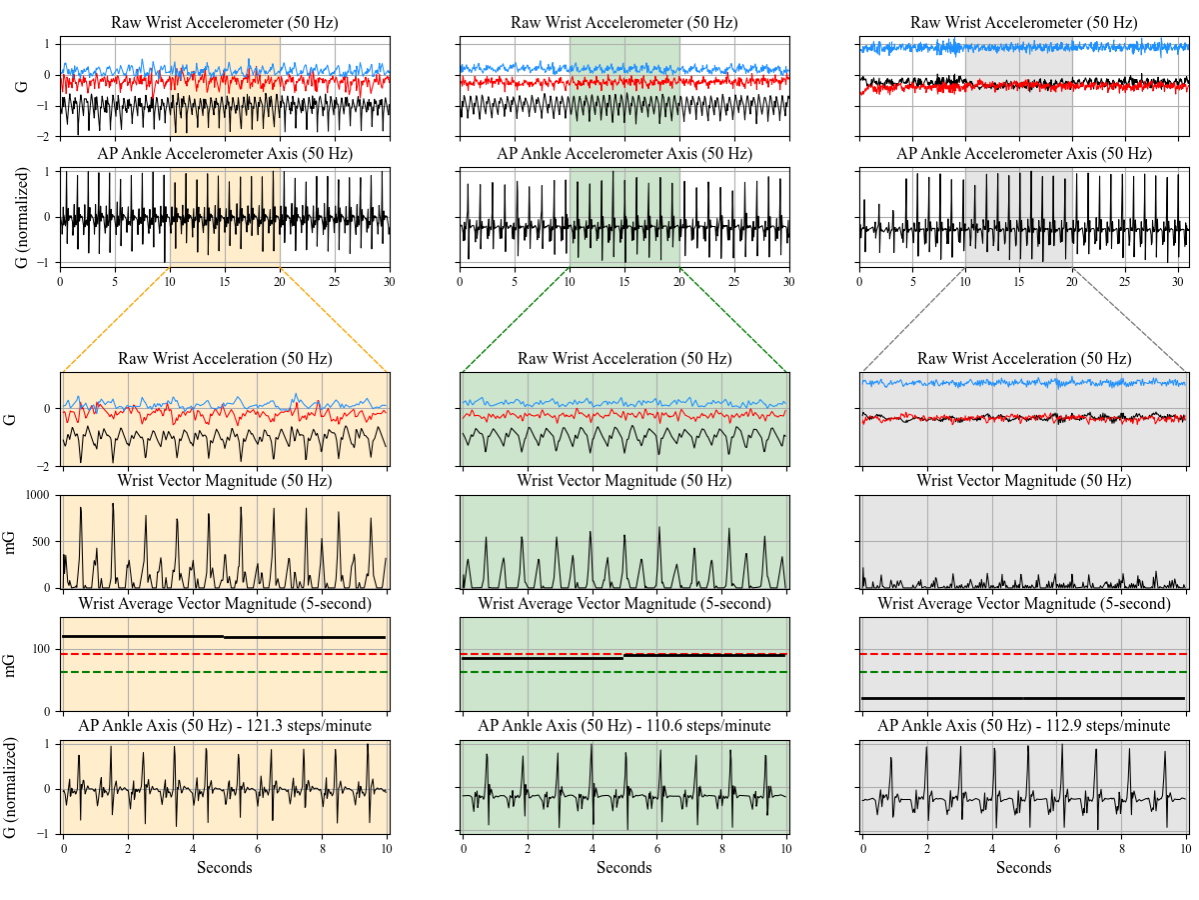
Transient changes in wrist acceleration profiles during a period of walking in a single older adult without a neurodegenerative disease diagnosis (OA) participant. Each column reflects 1 of 3 different segments of walking detected using the ankle accelerometer. The column shading represents the wrist-derived intensity classification for that walking segment (orange=moderate-to-vigorous physical activity (MVPA); green=light; gray=sedentary). The rows show data from the wrist or ankle accelerometers at various stages of data processing. The y-axis scales are consistent within all rows. Acceleration values have been normalized to values between -1 and 1 in the second and final rows (raw anteroposterior axis ankle acceleration). In the fifth row, green and red dashed lines represent the light and moderate intensity cut points, respectively, and the solid black lines are the average vector magnitudes (AVM) in 5-second epochs. Note that 5-second epochs were used in this figure for illustrative purposes due to the short duration of data segments in contrast to the 15-second epochs used in the analyses. AP: anteroposterior axis acceleration.

**Figure 3:**
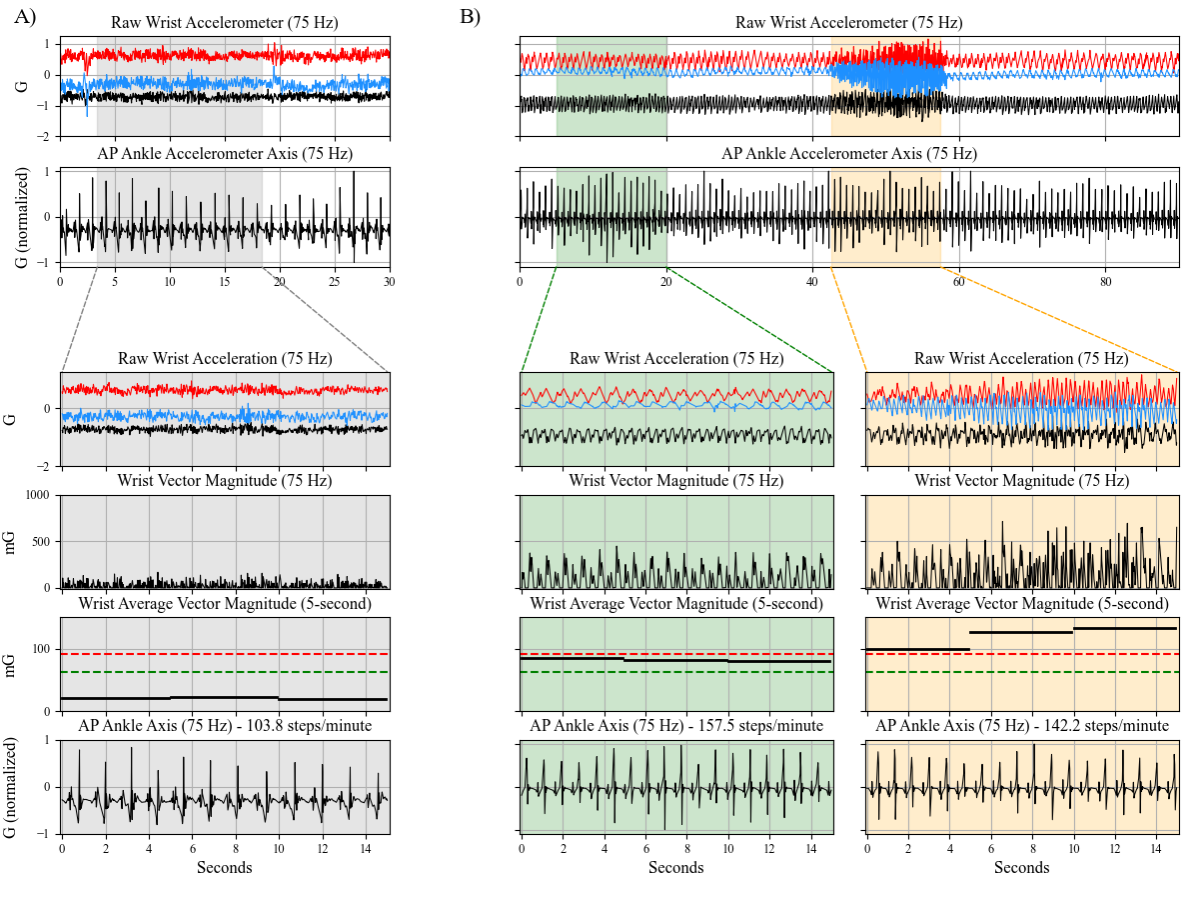
Transient changes in wrist acceleration profiles during a period of walking with a gait aid (A) and with tremor (B). Each column reflects 1 of 3 segments of walking detected using the ankle accelerometer for the 2 participants (left FTD1; right PD1). The column shading represents the wrist-derived intensity classification for that walking segment (orange=moderate-to-vigorous physical activity (MVPA); green=light; gray=sedentary). The rows show data from the wrist or ankle accelerometers at various stages of data processing. The y-axis scales are consistent within all rows. Acceleration values have been normalized to values between -1 and 1 in the second and final rows (raw anteroposterior axis ankle acceleration). In the fifth row, green and red dashed lines represent the light and moderate intensity cut points, respectively, and the solid black lines are the average vector magnitudes (AVM) in 5-second epochs. Note that 5-second epochs were used in this figure for illustrative purposes due to the short duration of data segments in contrast to the 15-second epochs used in the analyses. AP: anteroposterior axis acceleration.

The 3 participants chosen to illustrate instances that can lead to dissociation between wrist and ankle activity during free-living walking are highlighted in Figures 2 and 3, with cases detailed below.

##### Effect of Cadence and ADLs on Arm Swing

Figure 2 features a participant near the 50th percentile of the percent of wrist-derived sedentary classification during LONG walks in the OA cohort (OA6). The participant is a 65-year-old female who walked with a median cadence of 115 spm and had 12.3% (253/2054) of the LONG walking epochs classified as sedentary. Segments (columns) shown have cadences of 121, 111, and 113 spm with decreasing arm swing amplitudes, leading to wrist-derived MVPA, light, and sedentary classifications, respectively. While the columns on the left and middle show typical arm swing kinematics, the column on the right contains a horizontal forearm position with the palm facing down. This position, which may be indicative of an activity such as pushing a shopping cart, eliminates arm swing and leads to a wrist-derived classification of sedentary.

##### Effect of Gait Aid Use

Figure 3A features the participant with the highest percent of wrist-derived sedentary classification during LONG walks of gait aid users (FTD1). The participant is a 74-year-old male who uses a walker when walking outside, walked with a median cadence of 105 spm, and had 49.3% (277/562) of the LONG walking epochs classified as sedentary. Data from this participant show walking at a cadence of 104 spm. Despite this cadence, which is above the estimated threshold for MVPA [10], using the walker eliminated arm swing and resulted in a wrist-derived classification of sedentary behavior. Notably, the stepping pattern seen with the ankle-worn accelerometer remains similar to what is seen in Figure 2, highlighting the consistency of ankle accelerometry during gait despite behavioral changes that affect wrist-derived activity.

##### Disease Features That Impact Arm Swing

Figure 3B features a participant living with PD who had the highest measured mRS score, indicating the greatest degree of disability or dependence in ADLs within the study cohort. This participant is a 71-year-old male who walked with a median cadence of 135 spm and had 56% (867/1547) of the LONG walking epochs classified as sedentary (the most of all participants). Both the data segments are shown with very fast cadences (158 spm and 142 spm, respectively). The first segment (green) shows reduced arm swing, which leads to a wrist-derived light intensity classification despite a cadence of 158 spm. The second segment (orange) shows a cadence of 142 spm and evidence of a 4.5 Hz pronation-supination tremor within the raw wrist accelerometry data, leading to a wrist-derived classification of MVPA.

## Discussion

### Principal Findings

This study challenges current best practice wrist accelerometer methods, with evidence that the wrist does not consistently measure the intensity of known walking bouts within older adults and people living with NDD. Specifically, results showed that wrist-derived activity intensity estimates measured during known periods of free-living walking that were captured using ankle accelerometry are highly variable and frequently underestimated within these cohorts. All participants measured a statistically lower proportion of wrist-derived activity than expected, with 80% (28/35) of participants showing clinically meaningful misclassification of walking as sedentary behavior from wrist-derived intensity estimates. Results also demonstrate that transient, behavioral, or individual factors causing reduced or altered arm swing can contribute to variability in wrist-derived activity intensity classification during walking. These findings have important implications for free-living PA intensity classification and provide evidence for the value of a multidevice model for accurate measurement of daily PA in older adults and people living with complex health conditions.

Walking is a ubiquitous, whole-body activity that occurs throughout the day. As such, accurately capturing and classifying the amount of time spent walking in free-living is critical to understanding daily PA and the impact of disease or disability on its volume and distribution. This includes whether an individual achieves important thresholds for a healthy lifestyle, such as meeting the 24-Hour Movement Guidelines [8]. These guidelines include recommendations for time spent in MVPA, light activity, and sedentary behavior, based on evidence that each intensity has independent impacts on morbidity and mortality [17,18,53]. Walking at one’s preferred pace represents moderate-intensity activity throughout the adult lifespan and for various health conditions [11,28,54,55]. As the energy cost and expenditure at a given speed increase with age, preferred gait speed slows to maintain a constant energy expenditure [54-56]. While participants’ preferred paces were not explicitly measured in the current study, it was reasoned that constraining data analysis to longer periods of purposeful walking (ie, ≥60-second bout duration, ≤5-second break in between steps, termed “LONG” walks) increased the likelihood that participants were walking at their usual pace. Indeed, the median cadence of 107.3 (SD 10.9) spm across LONG walks included in this study is comparable to cadences at preferred walking speeds reported by others [54,57]. Importantly, this cadence aligns with the 100 spm threshold that has been shown to represent a moderate intensity in adults aged 21-85 years [10] and exceeds the 80 spm threshold that has been shown to represent a moderate intensity in people living with an NDD, specifically PD [28].

Despite confining the present analyses to continuous, long walking periods likely to reflect moderate-intensity activity, wrist-derived intensity estimates resulted in an average across participants of 22.9% of all LONG walking epochs being classified as sedentary (up to 56% (867/1,547) within participants) and an additional 27.7% being classified as light intensity (up to 63.7% (422/663) within participants). Even after stratifying sedentary behavior from light or MVPA, statistically and clinically meaningful underestimations of wrist-derived PA intensity were found in all participants. Free-living behavior has been shown to be dominated by short, fragmented, or sporadic bursts of walking [58,59] that are shorter than commonly used intensity classification epoch lengths. Including “short” bouts of walking when assessing activity intensity is likely to result in the total underestimation of activity intensity being greater than the underestimation found in this study when reliant on a single wrist-worn accelerometer. For example, if a 5-second walking bout occurs in a 15-second epoch used to classify activity intensity, the wrist-derived AVM for that epoch will be more representative of the longer period of inactivity (10 seconds) than activity (5 seconds). In this sample, “short” bouts of walking were substantial, making up an average of 72% of a person’s total walking time. Misclassification of continuous walking periods as sedentary behavior has important implications for clinical interpretation, PA prescription and monitoring at the individual level, and when looking within larger cohort studies, to further elucidate the impacts of engaging in activities of specific intensities on various health outcomes in older adults and people living with complex health conditions.

Although wrist-derived intensity estimates resulted in half of the walking time being misclassified as sedentary behavior or light activity, large variability between participants was evident during LONG walks (eg, 1.7% [31/1805 epochs] to 56% [867/1547 epochs] of walking time classified as sedentary). When grouped according to the percentage of sedentary time classified during LONG walks, statistically significant differences in demographics and behavior emerged between the upper and lower quartiles, including age and variability in the overall amount of arm acceleration during walking (AVM CoV). Notably, the upper quartile was older compared to the lower quartile (a mean difference of 7.4 years), which may have resulted in slower gait speeds [60] and reduced arm swing [61], leading to lower absolute wrist accelerometer output for a given energy expenditure and an underestimated activity intensity. Furthermore, the increased wrist AVM CoV in the upper quartile for sedentary time may be impacted by the composition of the group being predominantly people living with NDD (8/9, 89%) and including a participant who used a gait aid (compared to 44% (4/9) of the participants living with NDD and no gait aid users in the lower quartile). The use of some gait aids effectively immobilizes the wrist during walking (eg, walkers) or reduces arm swing (eg, canes), and motor symptoms or impairments common to NDD populations can impact arm motion (eg, upper limb hypokinesia in PD or weakness in ALS, muted arm swing in CVD) [62,63]. Wrist-based activity estimates become increasingly variable if motor symptoms fluctuate throughout the day or if gait aids are required only in certain situations or environments. All things considered, age differences, symptoms or features of the disease, and gait aid use may be considered key factors explaining the observed wrist-derived intensity differences during walking between and within people.

Additionally, this study illustrated that other transient behaviors associated with reduced arm swing during walking, such as concurrent engagement in ADLs, can contribute to the variability in wrist-derived activity intensity classification during walking within and between participants. As these variables can change on short timescales or occur unpredictably throughout the day, they cannot be accounted for systematically within the measurement approach. Applicable to participants of all health statuses, the simultaneous performance of other ADLs with walking, such as pushing a shopping cart or holding a grocery bag, will reduce arm swing and therefore acceleration amplitudes. As seen in Figure 3A, changes in wrist posture or reductions in arm movement can cause dissociation between movement measured at other body segments (eg, the ankle) and the wrist, leading to inaccurate wrist-derived activity intensity estimates. While the dynamic nature of these behaviors does not cause a substantial change in the ankle-derived accelerometer output, they are a primary limitation when using wrist-based accelerometry.

Beyond the person-specific factors that affect variability in wrist-derived activity intensity estimates during walking, it is also useful to examine the limitations inherent to the wrist accelerometry method. This study used cut points to estimate activity intensity from the wrist. While there is no consensus on best practices [64], this method is among the most common. Cut points facilitate the classification of wrist movement into activity intensity categories based on predefined activity count (AVM) thresholds. Although there is value in the ease of use and application to large population studies as a quick, low-burden way to track free-living activity, cut points are specific to the age, fitness, and health status (among other variables) of the sample in which they were developed [65,66]. Applications to dissimilar populations or confounding factors such as the presence of disease affect the validity of this approach. This study’s inclusion criteria of participants aged 65 years or older limited the effect of age on the selected cut points [44]. However, without individual calibration, the heterogeneous nature of the sample highlights the inherent limitations of using cut points to quantify PA.

The dissociation between wrist-derived activity intensity estimates during periods of moderate-intensity walking detected using ankle accelerometry supports the need for improved activity intensity classification, which may be addressed using a multidevice approach that considers the use of multiple wear locations (multinodal) and specific sensor types (multimodal) [23]. Adopting a multinodal setup, where devices are worn on different body segments (eg, wrist and ankle devices), accounts for periods of whole-body activity when the arm movement may not reflect overall energy expenditure (eg, using a gait aid), and allows for additional activities to be captured that may otherwise be missed when relying solely on a wrist-worn device (eg, cycling). Using both upper and lower limb devices may be particularly important in older adults and people living with NDD because the ankle accelerometer is a more sensitive measurement tool for gait assessment in people with slower gaits [67] or reduced arm swing. It may also be valuable to include multiple, specific sensor types that can measure different physiological signals. For example, continuous electrocardiography can be added to a setup that already includes an upper and lower limb accelerometer and has been shown to outperform proxy measures of heart rate obtained via photoplethysmography [68]. Including continuous electrocardiography in a multidevice approach can improve free-living activity intensity estimates using the well-established relationship between heart rate and energy expenditure [69], with the added benefit of movement context from the limb-worn accelerometers. Recent work has demonstrated that such a multimodal and multinodal approach is well-tolerated in older adults and a cohort of people living with CVD and NDD over a week-long wear period [23].

### Conclusions

In conclusion, this work has shown that a single, wrist-worn accelerometer produces highly variable activity intensity estimates during walking. This finding suggests that wrist-based accelerometry may be an unreliable tool for classifying activity intensity, specifically of walking, in older adults and people living with NDD. Ongoing work focuses on the use of multiple devices and sensors to better account for behaviors or disease-related features that may transiently or chronically reduce arm activity during walking and other activities. A multisensor approach has the potential to better characterize intra- and interindividual variance and more robustly quantify and classify free-living PA intensity. Improvements in activity intensity classification have implications for activity prescription and treatment monitoring for interventions designed to counteract age- or disease-related declines in function and quality of life.

## Abbreviations

AD/MCI: Alzheimer disease or amnestic mild cognitive impairment
ADL: activity of daily living
ALS: amyotrophic lateral sclerosis
AVM: average vector magnitude
CoV: coefficient of variation
CVD: cerebrovascular disease
FTD: frontotemporal dementia
HANDDS-ONT: Health in Aging and Neurodegenerative Diseases and DementiaS in Ontario
MoCA: Montreal Cognitive Assessment
mRS: modified Rankin Scale
MVPA: moderate-to-vigorous physical activity
NDD: neurodegenerative disease
OA: older adults without a neurodegenerative disease diagnosis
OA-NDD: older adults living with a neurodegenerative disease
ONDRI: Ontario Neurodegenerative Disease Research Initiative
PA: physical activity
PD: Parkinson disease
ReMiNDD: Remote Monitoring in Neurodegenerative Disease
spm: steps per minute

## Data Availability

ONDRI data—including but not limited to the data in this study—will be accessible through the Ontario Brain Institute (https://braininstitute.ca/) to qualified researchers. Please see the Ontario Brain Institute website for information on when the data will be released and how to access the data [70].
